# Radiomics-Based Prediction of Future Portal Vein Tumor Infiltration in Patients with HCC—A Proof-of-Concept Study

**DOI:** 10.3390/cancers14246036

**Published:** 2022-12-08

**Authors:** Fabian Stoehr, Roman Kloeckner, Daniel Pinto dos Santos, Mira Schnier, Lukas Müller, Aline Mähringer-Kunz, Thomas Dratsch, Sebastian Schotten, Arndt Weinmann, Peter Robert Galle, Jens Mittler, Christoph Düber, Felix Hahn

**Affiliations:** 1 Department of Diagnostic and Interventional Radiology, University Medical Center of the Johannes Gutenberg University Mainz, 55131 Mainz, Germany; 2 Institute of Interventional Radiology, University Hospital Schleswig-Holstein—Campus Luebeck, 23562 Luebeck, Germany; 3 Institute of Diagnostic and Interventional Radiology, University Hospital Frankfurt, 60590 Frankfurt, Germany; 4 Department of Diagnostic and Interventional Radiology, University Hospital of Cologne, 50937 Cologne, Germany; 5 Institute of Diagnostic and Interventional Radiology and Neuroradiology, Helios Dr. Horst Schmidt Kliniken Wiesbaden, 65199 Wiesbaden, Germany; 6 Department of Internal Medicine I, University Medical Center of the Johannes Gutenberg University Mainz, 55131 Mainz, Germany; 7 Department of General, Visceral and Transplant Surgery, University Medical Center of the Johannes Gutenberg University Mainz, 55131 Mainz, Germany

**Keywords:** carcinoma, hepatocellular, portal vein infiltration, radiomics, texture analysis, precision medicine

## Abstract

**Simple Summary:**

Portal vein infiltration (PVI) is a complication of HCC with critical impact on further patient management as systemic therapies are recommended once PVI is diagnosed. In our study, we matched 44 patients with HCC who developed PVI in the course of disease with no CT-detectable PVI at initial diagnosis to the same number of patients who never developed PVI during follow-up, but showed the same conventional tumor traits (size and number of lesions, growth type, contrast enhancement pattern, etc.). Using LASSO regression, radiomics feature analysis showed a sensitivity and specificity of 0.78 to detect the occurrence of PVI in the validation set. Therefore, an additional radiomics evaluation at initial diagnosis could help to identify patients benefiting from a closer surveillance.

**Abstract:**

Portal vein infiltration (PVI) is a typical complication of HCC. Once diagnosed, it leads to classification as BCLC C with an enormous impact on patient management, as systemic therapies are henceforth recommended. Our aim was to investigate whether radiomics analysis using imaging at initial diagnosis can predict the occurrence of PVI in the course of disease. Between 2008 and 2018, we retrospectively identified 44 patients with HCC and an in-house, multiphase CT scan at initial diagnosis who presented without CT-detectable PVI but developed it in the course of disease. Accounting for size and number of lesions, growth type, arterial enhancement pattern, Child–Pugh stage, AFP levels, and subsequent therapy, we matched 44 patients with HCC who did not develop PVI to those developing PVI in the course of disease (follow-up ended December 2021). After segmentation of the tumor at initial diagnosis and texture analysis, we used LASSO regression to find radiomics features suitable for PVI detection in this matched set. Using an 80:20 split between training and holdout validation dataset, 17 radiomics features remained in the fitted model. Applying the model to the holdout validation dataset, sensitivity to detect occurrence of PVI was 0.78 and specificity was 0.78. Radiomics feature extraction had the ability to detect aggressive HCC morphology likely to result in future PVI. An additional radiomics evaluation at initial diagnosis might be a useful tool to identify patients with HCC at risk for PVI during follow-up benefiting from a closer surveillance.

## 1. Introduction

Hepatocellular carcinoma (HCC) is the most frequent primary liver cancer and the fourth most common cause of cancer-related death [1,2]. According to the Barcelona clinic liver cancer (BCLC) classification, the prognosis of HCC is stage-dependent and patients with an advanced HCC have only limited therapeutic options [3].

Macrovascular tumor infiltration of portal and/or liver veins is a common and severe complication in patients with HCC. It occurs in up to 40% in patients with HCC and leads to a significantly poorer overall survival [4,5,6,7,8]. This is especially true for portal vein infiltration (PVI), accounting for about 90% of macrovascular tumor infiltration [9]. Plausibly, the greater the extent of PVI, the worse the prognosis becomes. It is noteworthy, however, that even segmental or subsegmental PVI leads to a dismal prognosis [7].

Although PVI has critical impact on the outcome and management of patients with HCC, an accurate diagnosis in clinical routine is often difficult. In particular, segmental or sub-segmental invasion is often missed at first occurrence and only diagnosed in retrospect [10,11]. As HCCs with PVI often show a fast progression, early diagnosis is important in order to avoid missing the chance of a treatment change, especially in the light of novel immunotherapeutic agents such as atezolizumab and bevacizumab [12,13,14]. If PVI is diagnosed, patients should be staged as BCLC C, and are usually not candidates for curative treatment options [3,15].

Thus, a method to non-invasively identify patients with HCC at risk of developing PVI more precisely, or at an earlier timepoint, would allow for better stratification of patients to the appropriate therapy. On this issue, converting imaging information into quantitative features and detecting disease-specific characteristic patterns is at the heart of radiomics [16,17]. Moreover, comprehensive radiomics-based quantification of tumor tissue has gained interest in oncologic imaging by providing an opportunity for improving decision support [18,19].

Against this background, we aimed to (1) investigate if there are quantifiable radiomics feature differences in HCC developing PVI vs. non-PVI, which would allow us (2) to develop a radiomics-based prediction model for PVI development in patients with HCC.

## 2. Materials and Methods

### 2.1. Study Design and Patient Recruitment

This retrospective cohort study conforms to the ethical guidelines of the 1975 Declaration of Helsinki and was approved by the responsible ethics committee (permit number 2018-13619). Patient data were collected from a clinical database installed in 1998 at our university medical center [20]. Inclusion criteria were as follows: (1) age > 18 years, (2) HCC diagnosis between 2008 and 2018, (3) no prior treatment, (4) in-house, multiphase contrast-enhanced CT scan at initial diagnosis, and (5) PVI observed during follow-up. We used the same database to select a control group of treatment-naïve HCC patients at random who met criteria (1)–(4) but never developed PVI. Follow-up ended 31 December 2021.

### 2.2. CT Examinations and Imaging Analysis

CT scans were performed using a Philips iCT or Philips Brilliance scanner (Philips Healthcare, Best, The Netherlands) in late arterial, portal venous, and delayed phase. Reconstruction mode was iDose level 3 with a Standard (B) filter and a 512 × 512 matrix. CT X-ray tube voltages were 80 kV for late arterial imaging and 120 kV for portal venous and delayed phase imaging. Extraction and subsequent analysis of imaging data was performed using our picture archive and communication system (PACS) (Sectra, Linköping, Sweden).

CT images were retrospectively analyzed by three board-certified radiologists with longstanding experience in liver imaging (RK, SS, FH). If necessary, differentiation between bland and tumor thrombus was performed by using established imaging criteria [11]. Doubtful imaging studies were analyzed in a second review by two of the radiologists until a consensus view was built.

### 2.3. Segmentation and Texture Analysis

**Figure 1:**
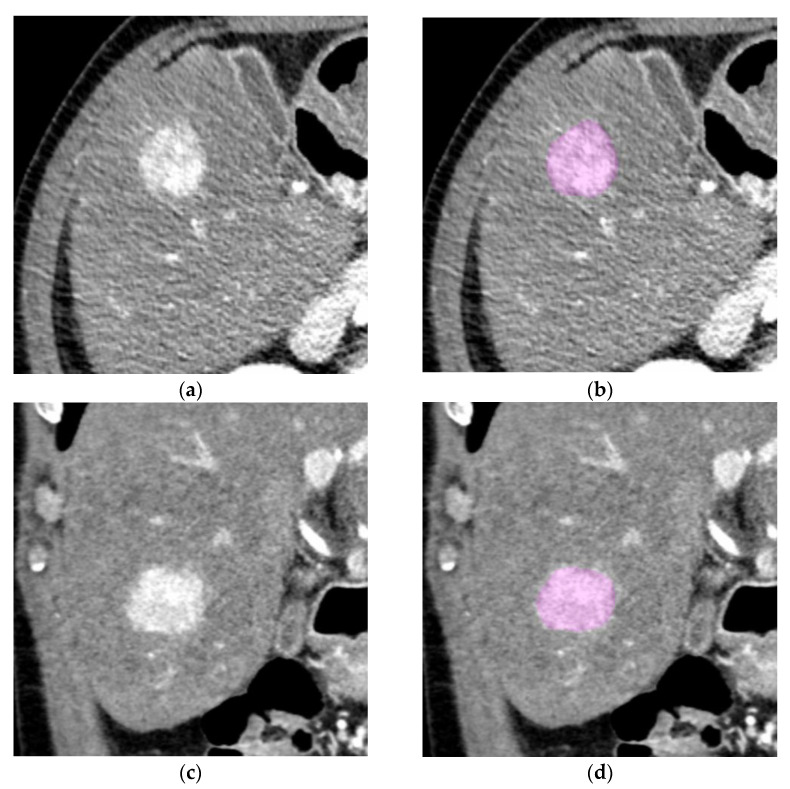
ROI segmentation of a tumor in the late arterial phase. (**a**,**c**) Exemplary axial and coronal slices of a sample tumor without PVI before segmentation; (**b**,**d**) exemplary axial and coronal slices of a sample tumor without PVI after segmentation.

ROI segmentation and texture analysis was performed using LifeX software (www.lifexsoft.org, Orsay, France) [21]. For data extraction and feature analysis, three-dimensional ROIs were drawn for the whole tumor volume. In case of multifocal hepatic tumors at initial diagnosis, the leading tumor was selected for segmentation. Segmentation was manually performed in both arterial and portal venous phase by two readers (FS and MS) after a consensus briefing on tumor margins (Figure 1). Regarding settings for radiomics analysis, a priori parameters included voxel normalization to 2 mm in all axes as well as focusing on the Hounsfield range between −64 and 448 Hounsfield units with 128 bins. Overall, 52 texture features were computed in each of the two CT phases for a total of 104 texture features.

### 2.4. Statistical Analysis

Statistical analyses were performed using R 4.0.3 (A Language and Environment for Statistical Computing, R Foundation for Statistical Computing, https://www.R-project.org; last accessed on 31 October 2022). Using propensity score matching accounting for size and number of intrahepatic lesions, growth type, non-rim arterial enhancement pattern, Child–Pugh stage, alpha-fetoprotein (AFP) levels, and subsequent therapy, we matched an equal number of patients with HCC who did not develop PVI to those with PVI in the course of disease. The R package “matchIt” was used for propensity score matching. LASSO regression was performed using the R package “glmnet” with binomial logistic regression (https://CRAN.R-project.org/package=MatchIt, https://CRAN.R-project.org/package=glmnet, last accessed on 31 October 2022). Pearson’s correlation was computed to identify redundant features; in case of highly correlated features (r > 0.9), one feature was dropped. For the prediction model, the patients were randomly split up in an 80%/20% ratio into a training and a holdout validation set. The LASSO regression was built using a cross-validation approach upon the training set, testing of the model was performed on the independent validation set. *p* values less than 0.05 were considered statistically significant.

## 3. Results

### 3.1. Baseline Characteristics

Employing the inclusion criteria set forth in the previous section, 44 patients were included in this study who initially showed no signs of portal vein infiltration but developed PVI later in the course of disease. As a control group, 213 patients with no signs of PVI, neither in initial nor in follow-up imaging, were randomly selected. Median follow-up time of patients without PVI was 680 days (IQR: 270–1240 days). In the cohort of HCC patients developing PVI only in the course of disease, median time to PVI was 209 days (IQR: 84–419 days). Using propensity score matching, the 44 tumors of the patients who developed PVI in the course of the disease were matched to 44 patients who never developed PVI. Detailed baseline characteristics of the matched groups are provided in Table 1.

### 3.2. Feature Selection and Prediction Model Using LASSO Regression

**Figure 2:**
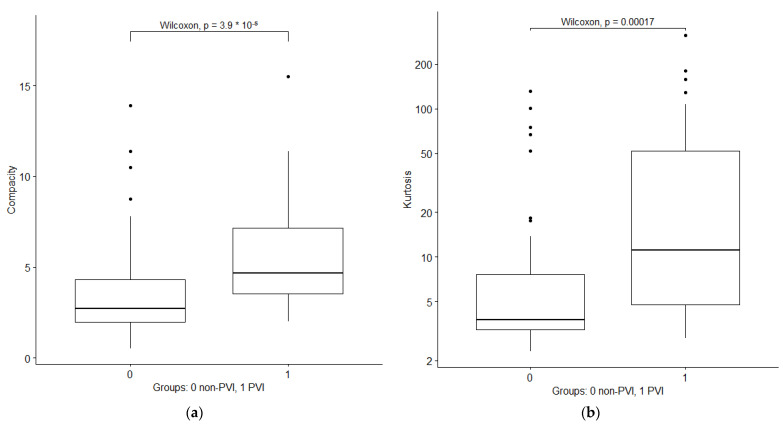
Boxplots of first order radiomics features compacity and kurtosis in portal venous phase in patients without and with PVI development. (**a**) Compacity; (**b**) kurtosis.

After dropping redundant features with high correlation, a total of 47 radiomics features out of 104 initial features remained (Supplementary Table S1). Among first order features, the most significant features were shape compacity and kurtosis, indicating more extreme outliers and a less sphere-like volume of tumors with future PVI. Boxplots of the two features are depicted in Figure 2.

**Figure 3:**
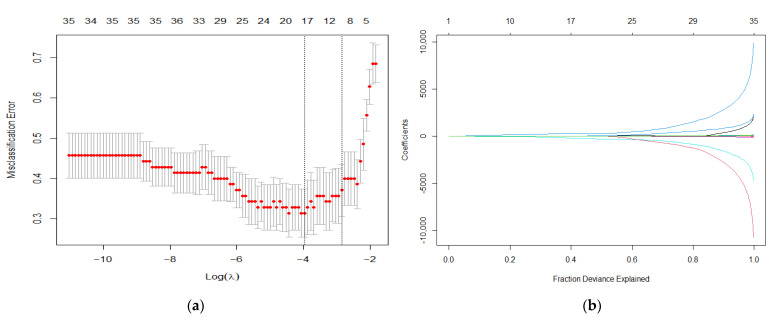
Presentation of the radiomics feature selection. (**a**) Lambda chosen via minimum criteria using 10-fold cross validation; (**b**) fraction deviance explained of the LASSO coefficients.

After a random 80:20 split of the dataset in a training and a validation set, and using LASSO regression analysis on the training set, a 10-fold cross-validation approach yielded a minimum lambda of 0.019 (Figure 3). Applying the fitted model to the independent validation dataset, sensitivity was 0.78 and specificity was 0.78. The radiomics model with its coefficients is provided in the supplement (Supplementary List S1, the contingency tables of the regression on the training and validation set are depicted in Table 2.

## 4. Discussion

Our results indicate an added value of radiomics analysis at initial diagnosis in the detection of future portal vein infiltration in patients with HCC. While differences in tumor contrast enhancement, tumor shape, and tumor size have been previously proposed as risk factors for PVI development, quantitative tissue characteristics helped predict infiltration when matching for those conventional tumor traits.

Ideally, nodular HCCs show a strong arterial phase hyper-enhancement, followed by early washout in the portal venous and delayed phase [22]. Compared to nodular HCCs, HCCs with PVI are often ill-defined and show diffuse, infiltrative growth patterns, making it sometimes challenging to detect them against the cirrhotic liver parenchyma [23]. Regarding their contrast enhancement, HCCs with PVI often show inhomogeneous contrast enhancement on arterial phase and portal venous/delayed phase [23,24]. These conventional tumor traits have been associated with portal vein infiltration; however, they rely on qualitative assessments by the reader.

Moreover, HCCs with PVI have been associated with a larger tumor size regarding both tumor volume and number of lesions [12,13]. Together with the above-mentioned infiltrative growth patterns, HCCs with PVI often display a large intrahepatic tumor burden affecting major parts of the liver, not allowing for curative treatment [3].

In our study, we showed that a radiomics model measuring quantitative intralesional tissue characteristics might help to distinguish PVI development in patients with HCC. This refers to the so-called tumor heterogeneity, and has recently gained interest in oncologic imaging [18,25]. The term heterogeneity covers a broad range of histological features, including tumor grading, angiogenesis, necrosis, cellularity, etc., which can be quantified by radiomics measurements [18,26]. Most importantly, as recent studies could show, heterogeneity is a relevant attribute associated with poorer prognosis or clinically relevant mutations [18,19,27]. Thus, it has been hypothesized that tumor heterogeneity might be an expression of a more aggressive tumor biology [18].

Regarding our results, and considering that PVI was associated with tumor heterogeneity, PVI could be a phenotypical manifestation of adverse tumor biology. Thus, the noninvasive assessment of tumor heterogeneity could further optimize treatment stratification by selecting patients more individually [18,26]. Recent studies have shown positive results in predicting microvascular invasion in HCC using radiomics approaches: Xu et al. incorporated clinico-radiologic and radiomics parameters to achieve an area under the curve of 0.91 in their training and 0.89 in their test set for prediction of microvascular infiltration in a cohort of surgically resected HCC patients [28], while Shan et al. investigated radiomics models of both tumoral and peritumoral tissue to predict early recurrence of HCC [29]. Since microvascular invasion is a very strong predictor for tumor recurrence after curative treatment [30], these studies might impact future patient selection. Moreover, radiomics has shown the potential to predict HCC grading preoperatively [31].

As things stand today, there are plenty of staging systems rating patient survival mainly based on clinical and imaging data, most notably the BCLC classification [32,33]. The BCLC classification recommends transarterial treatment for intermediate stage (BCLC B) and systemic therapy for advanced stage HCC with PVI (BCLC C) [3,34]. However, since these subgroups are partly overlapping, it is very difficult to predict which patient might profit and which one might not profit from a particular therapy [35]. Moreover, HCCs with PVI display high interindividual genetic tumor heterogeneity with different prognoses, and there is increasing evidence that a more aggressive treatment might be beneficial for selected patients [12,26]. Thus, current staging systems could profit by taking individual tumor behavior more into account [26,36]. By incorporating radiomics features, this would allow for a more precise stratification of patients to the optimal treatment [37]. 

### Limitations

As a preliminary study, it has several inherent limitations. Most importantly, the study was conducted retrospectively at a single center. As the images were acquired and reconstructed using two CT scanner types by one vendor, the reproducibility of our model under different imaging settings has not been tested. Therefore, our results may not necessarily be transferrable to other care centers and their patient collectives. In radiomics analyses, reproducibility is related to various factors including imaging acquisition protocols, reconstruction algorithms used, methods of segmentation, and methods for extracting imaging features—due to the lack of standardization among institutions, generalizability has been a problem [17,26,37,38]. Thus, although we used a holdout patient cohort not used for training as validation, external validation of the model has to be performed in order to prove its stability and reproducibility.

Furthermore, segmentation of tumor ROIs was performed manually, making it a highly time-consuming and potentially error-prone procedure. However, there are several studies showing how semi-automatic and fully automatic ROI detection, especially using deep learning methods, can be successfully used to improve expenditure of time as well as accuracy [39,40,41]. Therefore, automatization is likely to not only simplify but also objectify the segmentation procedure. However, these automated segmentations have not become part of clinical routine in radiological tumor assessments.

In the period of patient inclusion between 2008 and 2018, diagnosis of HCC was routinely made on cross-sectional imaging using established criteria; histological sampling of the tumor was not necessarily performed [42]. Moreover, patients in this study were treated with different types of therapies after initial diagnosis. Thus, it cannot be ruled out that treatment-related side effects affected the tumor biology in an adverse way, e.g., in the case of incomplete ablation, which might have favored subsequent PVI [43,44].

## 5. Conclusions

Our study successfully demonstrates a proof-of-concept radiomics model predicting future PVI in patients with HCC at initial diagnosis. As PVI leads to a dismal prognosis and is often missed in the clinical routine at first occurrence, an additional radiomics evaluation recognizing red flags for patients at risk for PVI during follow-up will help to identify those benefiting from a closer surveillance. Thus, the ability of radiomics to detect aggressive HCC morphology might provide one additional aspect in patient evaluation and stratification.

## Figures and Tables

**Table 1:** Baseline characteristics of the patient groups without and with future PVI [IQR interquartile range, AFP = alpha-fetoprotein, NASH = nonalcoholic steatohepatitis].

Parameter	Non-PVI Group (*n* = 44)	PVI Group (*n* = 44)	*p*-Value
**Age, years [IQR]**	65 [59–72]	71 [63–74]	0.05
**Number of lesions, n [IQR]**	3 [1–6]	4 [2–9]	0.59
**Size of lesions, mm, median [IQR]**	39 [28–56]	44 [32–68]	0.62
**Growth type**			
nodular, n	36	34	
diffuse, n	8	10	0.71
**Non-rim arterial enhancement pattern**			
hypervascular, n	23	25	
hypovascular, n	4	4	
mixed, n	27	15	0.90
**Child–Pugh stage**			
A, n	22	26	
B, n	22	17	
C, n	0	1	0.37
**AFP levels, ng/mL, mean [IQR]**	11,946 [16–22,316]	15,193 [38–43,866]	0.45
**Etiology**			
C2, n	18	21	
chronic hepatitis B, n	8	6	
chronic hepatitis C, n	12	10	
NASH, n	4	3	
unknown, n	2	4	0.83
Initial treatment *			
curative, n	10	8	
intra-arterial, n	33	35	
systemic, n	1	1	0.87

* Curative includes surgery and ablation, intra-arterial includes trans-arterial chemo-embolization and selective internal radiation therapy.

**Table 2:** Contingency tables of the radiomics model against the ground truth in the training and holdout validation set.

Training Set	No PVI Occurred	PVI Occurred
No PVI predicted	25 (71%)	6 (17%)
PVI predicted	10 (29%)	29 (83%)
**Holdout validation set**	**No PVI occurred**	**PVI occurred**
No PVI predicted	7 (78%)	2 (22%)
PVI predicted	2 (22%)	7 (78%)

## Data Availability

Data cannot be shared publicly because of institutional and national data policy restrictions imposed by the Ethics Committee of the Medical Association of Rhineland Palatinate, Mainz, Germany, since the data contain potentially identifying patient information. Data are available upon request for researchers who meet the criteria for access to confidential data.

## Supplementary Materials

The following supporting information can be downloaded at: https://www.mdpi.com/article/10.3390/cancers14246036/s1, Table S1: Radiomics features included in the analysis after dropping highly correlated features; List S1: Radiomics LASSO regression formula.
