# Supplementary material for: Radiomics-Based Prediction of Future Portal Vein Tumor Infiltration in Patients with HCC—A Proof-of-Concept Study
Source: Cancers (Basel). 2022 Dec 8;14(24):6036. doi: 10.3390/cancers14246036 (PMC9775514; doi:10.3390/cancers14246036)
Supplement: Supplementary file 1 [file cancers-14-06036-s001.zip › List S1_Radiomics LASSO regression formula.pdf]

Supplement List S1

Radiomics LASSO regression formula:

-1.960515e+00 + art_HISTO_Skewness*6.663120e-02 + art_SHAPE_Sphericity*(-1.417622e+01) + art_GLCM_Correlation*(-6.356373e-01) + art_GLRLM_LRLGE*2.018372e+01 + art_NGLDM_Coarseness*2.478649e+02 + art_NGLDM_Contrast*(-2.541552e+00) + art_GLZLM_SZE*8.944418e+00 + art_GLZLM_SZHGE*(-2.510421e-04) + art_GLZLM_LZHGE*3.579658e-07 + ven_CONVENTIONAL_HUmax*8.360941e-04 + ven_CONVENTIONAL_HUpeak.sphere.0.5mL*1.049836e-02 + ven_SHAPE_Sphericity*5.628418e+00 + ven_GLCM_Correlation*3.865242e-01 + ven_GLCM_Entropy_log2*3.462243e-02 + ven_GLZLM_SZE*4.410919e+00 + ven_GLZLM_SZLGE*2.678224e+02 + ven_GLZLM_LZHGE*4.970378e-07
