# Supplementary material for: Radiomics-Based Prediction of Future Portal Vein Tumor Infiltration in Patients with HCC—A Proof-of-Concept Study
Source: Cancers (Basel). 2022 Dec 8;14(24):6036. doi: 10.3390/cancers14246036 (PMC9775514; doi:10.3390/cancers14246036)
Supplement: Supplementary file 1 [file cancers-14-06036-s001.zip › Table S1_radiomics features included in the analysis after dropping highly correlated features.pdf]

Supplement Table S1

Radiomics features included in the analysis after dropping highly correlated features:

Late arterial phase	Portal venous phase
CONVENTIONAL_HUmin	CONVENTIONAL_HUmin
CONVENTIONAL_HUstd	CONVENTIONAL_HUstd
CONVENTIONAL_HUmax	CONVENTIONAL_HUmax
CONVENTIONAL_HUpeak.sphere.0.5mL	CONVENTIONAL_HUpeak.sphere.0.5mL
CONVENTIONAL_HUpeak.sphere.1mL	CONVENTIONAL_HUpeak.sphere.1mL
HISTO_Skewness	HISTO_Skewness
HISTO_ExcessKurtosis	HISTO_ExcessKurtosis
SHAPE_Sphericity	HISTO_Energy...Uniformity.
GLCM_Correlation	SHAPE_Sphericity
GLCM_Entropy_log2	SHAPE_Compacity
GLCM_Dissimilarity	GLCM_Energy...Angular.second.moment
GLRLM_LRLGE	GLCM_Correlation
GLRLM_RP	GLCM_Entropy_log2
NGLDM_Coarseness	GLCM_Dissimilarity
NGLDM_Contrast	GLRLM_LRLGE
GLZLM_SZE	NGLDM_Coarseness
GLZLM_SZLGE	NGLDM_Contrast
GLZLM_SZHGE	GLZLM_SZE
GLZLM_LZLGE	GLZLM_SZLGE
GLZLM_LZHGE	GLZLM_SZHGE
GLZLM_ZLNU	GLZLM_LZLGE
GLZLM_ZP	GLZLM_LZHGE
	GLZLM_GLNU
	GLZLM_ZLNU
	GLZLM_ZP
